# Factors contributing to the abundance and spatial distribution of the invasive intermediate host snail (*Pseudosuccinea columella)* in uMgungundlovu district, KwaZulu-Natal, South Africa

**DOI:** 10.1186/s12917-025-04703-1

**Published:** 2025-04-09

**Authors:** Mpumelelo Ian Hadebe, Tawanda Manyangadze, Chester Kalinda, Moses John Chimbari

**Affiliations:** 1 Discipline of Public Health and Nursing, College of Health Sciences, University of Kwazulu-Natal, Durban, 4000 South Africa; 2 Department of Geosciences, School of Geosciences, Disaster and Development, Faculty of Science and Engineering, Bindura University of Science Education, P.O. Box 1020, Bindura, Zimbabwe; 3 Bill and Joyce Cummings Institute of Global Health, University of Global Health Equity (UGHE), P.O. Box 6955, Kigali, 20093 Rwanda

**Keywords:** Diseases, Freshwater snail, Habitats, Physicochemical parameters

## Abstract

Fascioliasis is a parasitic disease commonly affecting cattle, goats, and sheep globally. Lymnaeidae snail species are important in the epidemiology and dispersal of fascioliasis since they are intermediate hosts of the *Fasciola* spp. Our study mapped at micro-geographical scale, the distribution and abundance of *Pseudosuccinea columella* in uMgungundlovu district, which is in the northern part of the KwaZulu-Natal province, and measured physicochemical parameters at potential transmission sites. The study examined the impact of physicochemical parameters and presence of other snail species on *P.columella* abundance and distribution in KwaZulu-Natal's uMgungundlovu district. Data were analyzed using R studio, a negative binomial mixed model, and various statistical tests, including the variance inflation factor and the Wilcoxon rank sum test. Overall, 1406 freshwater snails, distributed in 45 sampling sites. *Pseudosuccinea columella* (569) had a widespread coverage in 34 sites (75.6%) of them but not found at 11 sites. Water pH ranged between 6.60 ± 0.38 and 7.46 ± 0.15, while dissolved oxygen (DO) values varied across the sites. GLM analysis suggested that water pH had an influence on the abundance of *P.columella*. The intermediate host snail of *Fasciola*, *P. columella* is abundant and widely distributed across all the 7 municipalities in the uMgungundlovu district suggesting the need for increased snail monitoring to reduce its invasiveness and livestock productivity losses due to *Fasciola* infections.

## Introduction

Fascioliasis is considered as one of the most globally spread zoonotic parasitic diseases, which extends widely across a large variety of habitats in different geographic regions [51]. The global economic impact on the livestock sector is estimated to be approximately £2 billion annually [48]. These production losses can be attributed to decreased livestock productivity, liver condemnation, a lower carcass value, and livestock mortality due to fascioliasis [25, 31]. Fascioliasis is mostly caused by the hermaphroditic parasites *Fasciola hepatica* (Linnaeus, 1758) and *Fasciola gigantica* (Cobbold, 1855) [46, 50], affecting cattle, goats, sheep, wildlife and humans [36]. The epidemiology of the disease is linked to the ecological characteristics of the snail vectors involved in the transmission of the parasites [6, 28]. The presence and abundance of suitable intermediate host snails is a determinant of infection risk [1, 30]. Hadebe et al. [21] suggested that the distribution and abundance of the intermediate host snails are indicators of disease hotspots and are hence important to know for disease control.

Approximately 5000 snail species inhabit diverse habitats worldwide, with an estimation of 350 snail species considered as medically important [38]. Among them are lymnaeid snails (Gastropoda: Lymnaeidae) which are intermediate hosts of *F. hepatica*. Lymnaeid snails have been observed to have high ecological plasticity thus, allowing them to inhabit a wide range of ecosystems thus increasing the risks of fascioliasis transmission [51]. Emerging evidence suggests that several habitats previously inhabited by *Radix natalensis* (Krauss, 1848) *or Galba truncatula* (Muller, 1774) as the major intermediate host snails of *Fasciola* are being taken over by another snail, *Pseudosuccinea columella* (Say, 1817) [25]*. Pseudosuccinea columella* is an invasive freshwater snail that was introduced to several countries from North America [20]. After introduction, Fasciolosis increased in real conditions in New-Zealand [42, 43]. In Africa, it was first discovered in South Africa in the middle of the twentieth century, but is currently found throughout the continent [14, 15].

Numerous studies have recognized *Pseudosuccinea columella* as an important intermediate host to *Fasciola*. According to a study by Boray [9], the importance of this species in the life cycle of the parasite was documented for the first time. Studies by Cruiz-Reyes and Malek (1987) [13] corroborated that *Fasciola* was capable of developing within the host. More recent studies including Malatji et al. [26],Ngcamphalala et al. [33] have strengthened those claims by demonstrating the relatively wide distribution and adaptation of *P. columella* as a host which may affect the epidemiology of fascioliasis in different parts of the world.

Its wide distribution has increased the negative health effects on livestock and financial implications as it can serve as an intermediary host for both *F. hepatica* [16, 29, 30] and *F. gigantica* [20] compared to native *Fasciola* vector snails *R. natalensis* known to transmit *F. gigantica* [29] and *G. truncatula* known to be the main intermediate host of *F. hepatica* [4]. *Pseudosuccinea columella* is thought to transmit both *Fasciola* species in countries like South Africa because of the noted rise in the infection rate of both trematodes, which occurred concurrently with *P. columella's* entry into South Africa [25]. The increase in the invasion and colonization sites by this snail, although not extensively explored, may be due to the snail’s aquatic behaviour; its tolerance to extreme climatic conditions; and its reproductive superiority over most other lymnaeids [12, 14, 41].

In South Africa, *P.columella* has been found in slowly-running rivers and streams, areas with stagnant water, abundant vegetation and a muddy substratum [33]. *Pseudosuccinea columella* has also been observed in small man-made habitats such as dams, water tanks and drinking troughs for cattle [12], habitats that are characterized by different climatic, environmental, and water physicochemical parameters that may influence snail growth, reproduction, and survival [49]. Several studies have been done on the distribution and abundance of native intermediate host snails of fascioliasis [17, 24] compared to studies on *P. columella*. Knowledge of the spatial distribution and abundance of *P. columella* is important for determining the risk areas for both *F. hepatica* and *F gigantica* transmission in uMgungundlovu district KwaZulu Natal Province South Africa. This study mapped the distribution and abundance of *P. columella* in relation to environmental parameters.

## Methodology

### Study area

**Fig. 1:**
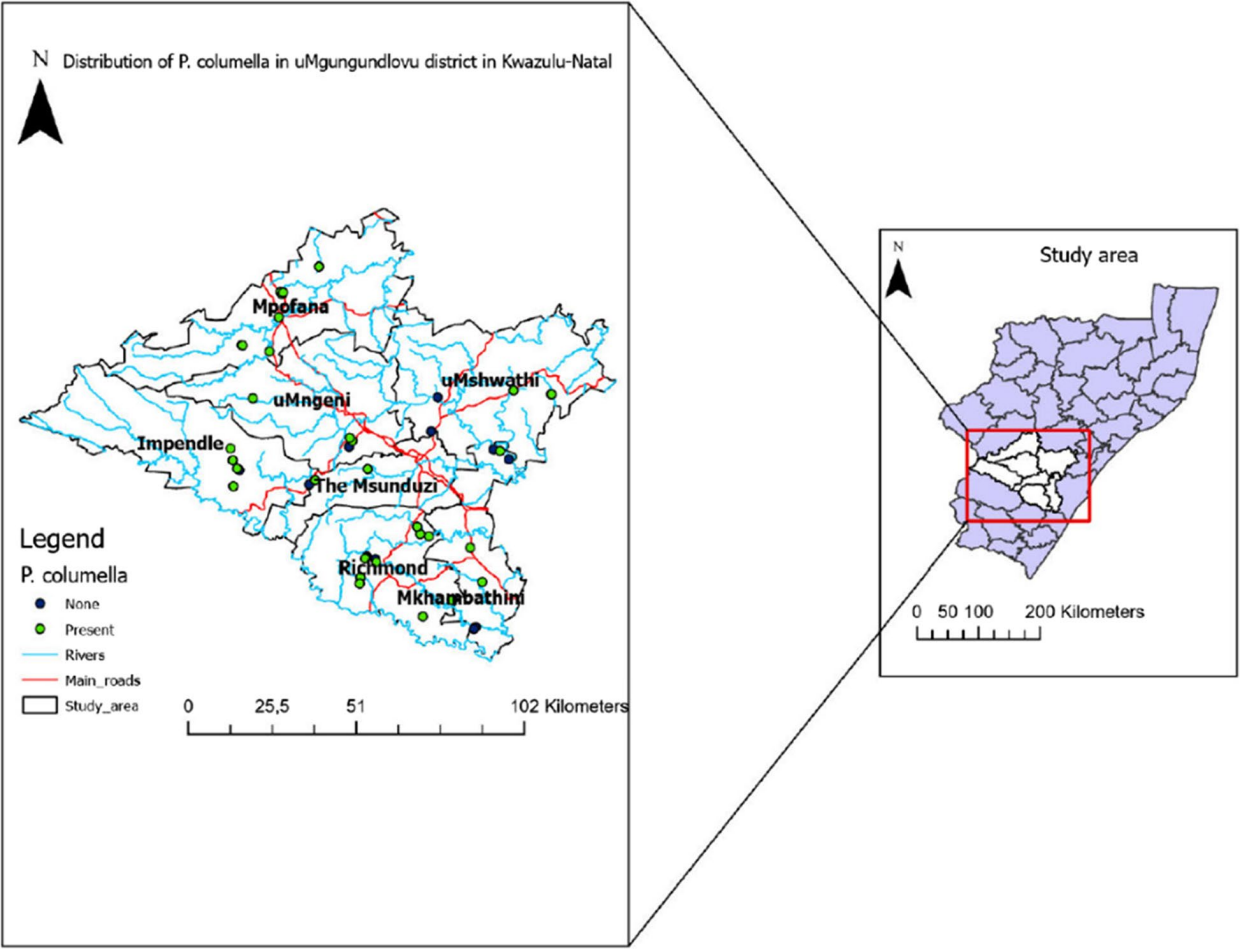
Map showing the sites with/without *P. columella* within the seven local municipalities of uMgungundlovu district

This study was conducted in the uMgungundlovu district, one of the 10 district municipalities located in KwaZulu-Natal. The uMgungundlovu district has a surface area of 9513 km^2^ and comprises seven local municipalities, namely: Msunduzi, Impendle, uMshwathi, Mkhambathini, Mpofana, Umngeni, and the Richmond (Fig. 1). These municipalities are primarily dominated by agricultural activities and livestock farming, which contributes significantly in the economy of the district. In 2017 the Umgungundlovu district recorded the largest provincial share in all the three categories (22,1% of number of farms, 24,2% of income and 19,6% of employment). eThekwini had the second largest share of income (12,7%) and employment (18,2%) while, Uthukela had the second largest share of number of farms (11,7%) [2]. It is therefore important to monitor the distribution of *Fasciola* intermediate host snails for fascioliasis as they may have a negative impact in livestock production resulting in loss of employment.

### Snail sampling

A stratified random cluster sampling procedure was used in selecting the water bodies for fresh water snail collection in this study. This was done by grouping all the identified freshwater bodies (using google maps and local knowledge) and sites from the municipalities into temporary and permanent water bodies. Also considered in sites selection was easy access to the water body.

Snail sampling was carried out at 45 sites within the seven municipalities by a team of three individuals. Two were responsible for scooping as described by [5] and hand picking at each site for 30 min while the third individual recorded the geographical positions using a hand held global positioning system (GPS) machine for each site. The captured snails from each site were counted and morphologically identified to species level using Brown and Kristensen’s shell morphological identification key [11], and returned to their natural habitats.

### Physicochemical parameters, climatic and environmental factors

A multi-probe meter (Hanna HI 9829 multiparameter) was used to measure the following water physico-chemical parameters; water pH, water pressure, water temperature, total dissolved oxygen, electrical conductivity, total dissolved solids, and salinity. Remote sensing was utilized to acquire the Normalized Difference Vegetation Index (NDVI), Normalized Difference Water Index (NDWI), Enhanced Vegetation Index (EVI), and precipitation were averaged during a two-week period (15 April–26 April 2024). Climate Engine: Cloud Computing of Climate and Remote Sensing Data (http://climateengine.org) provided access to all remote sensing data.

### Data analysis

The data collected from the field were recorded in Microsoft Excel spreadsheet and the data analysis was conducted in R studio version 4.4.0. The distribution of *P. columella* across the seven local municipalities was represented using a map created using ArcGIS pro. Wilcoxon rank sum test was performed to evaluate the effect of habitat type (permanent or temporary) on the abundance of snails. A negative binomial generalized linear mixed model in the ‘*glmmTMB*’ package was used to identify physicochemical parameters and environmental factors influencing the abundance of the snail species [10]. Variance inflation factor (VIF) was used to determine the relationships and collinearity between variables. Variables with VIF value of more than 5 indicated collinearity [27], hence was excluded from the analysis. We used Akaike’s Information Criterion (AIC) and negative log-likelihood values to compare models and chose the final models with the least AIC [52].

## Results

### The abundance and spatial distribution of *P. columella* in uMgugundlovu district

**Fig. 2:**
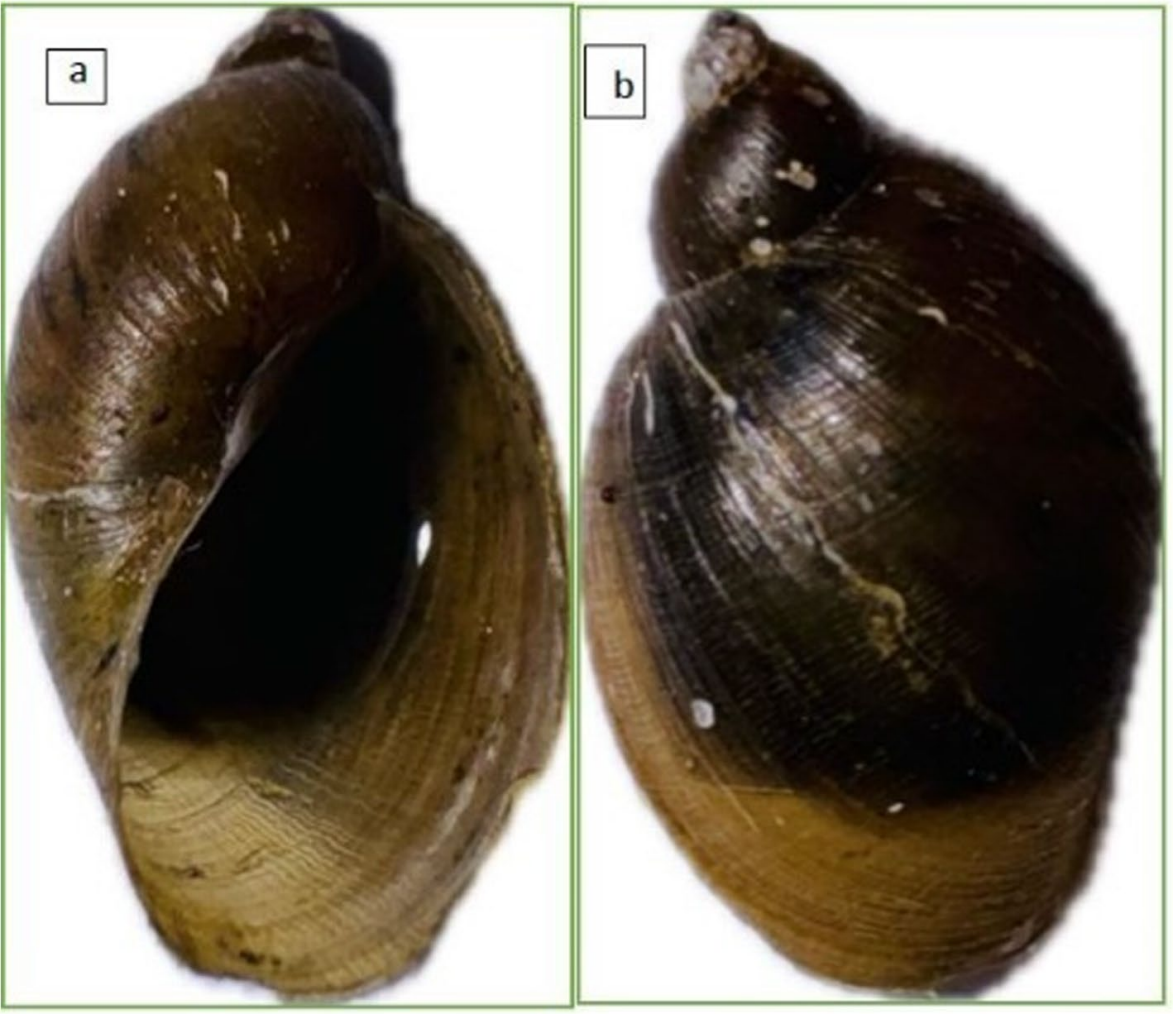
Images of *P. columella* collected from one of the 45 sites: **a** basal view, **b** Apical view

A total of 569 (From 1406) freshwater snails, distributed among 45 sites in 7 local municipalities of the uMgungundlovu district (Fig. 1) were morphologically identified as *P.columella* (Fig. 2). Among the 45 sampled sites, the invasive *P. columella* was present (indicated in green circles) in 34 (75.6%) scattered across seven municipalities. *P. columella* was not found at 11 sites (indicated in navy circles) (Fig. 1).

The shell of *P.columella* is distinguished by its elongated form, large opening (a), pointed spire, and delicate spiral threads (b) on its surface (Fig. 2).

Among the municipalities in which sampling was done, uMshwathi local municipality had the least *P. columella* abundance (*n* = 8, 1.4%), while Richmond local municipality had the highest abundance (*n* = 206, 36.2%). *P. collumella* was absent at 10 of the 45 sites spread across the 7 municipalities. Of the sites that had the snails, Richmond had the most (*n* = 9, 25.7%) sites where *P. columella* was found followed by Mpofana (*n* = 8, 22.9%); Mkhambathini, Impendle, and uMshwathi had (*n* = 4, 11.2%); uMngeni had (*n* = 3, 8.6%). On the other hand, only 2 (5.7%) sites in uMsunduzi had *P. collumella* (Table 1).

**Table 1:** Number of sites sampled in each local municipality and abundance of *Pseudosuccinea columella*

Municipality	Number of sites sampled	Sites with* P. columella*	*P. columella* Abundance
Richmond	11	9	206
Mpofana	9	8	150
Mkhambathini	8	4	40
Impendle	5	4	54
uMshwathi	5	4	8
uMngeni	4	3	96
uMsunduzi	3	2	15
**Total**	**45**	**34**	**569**

**Fig. 3:**
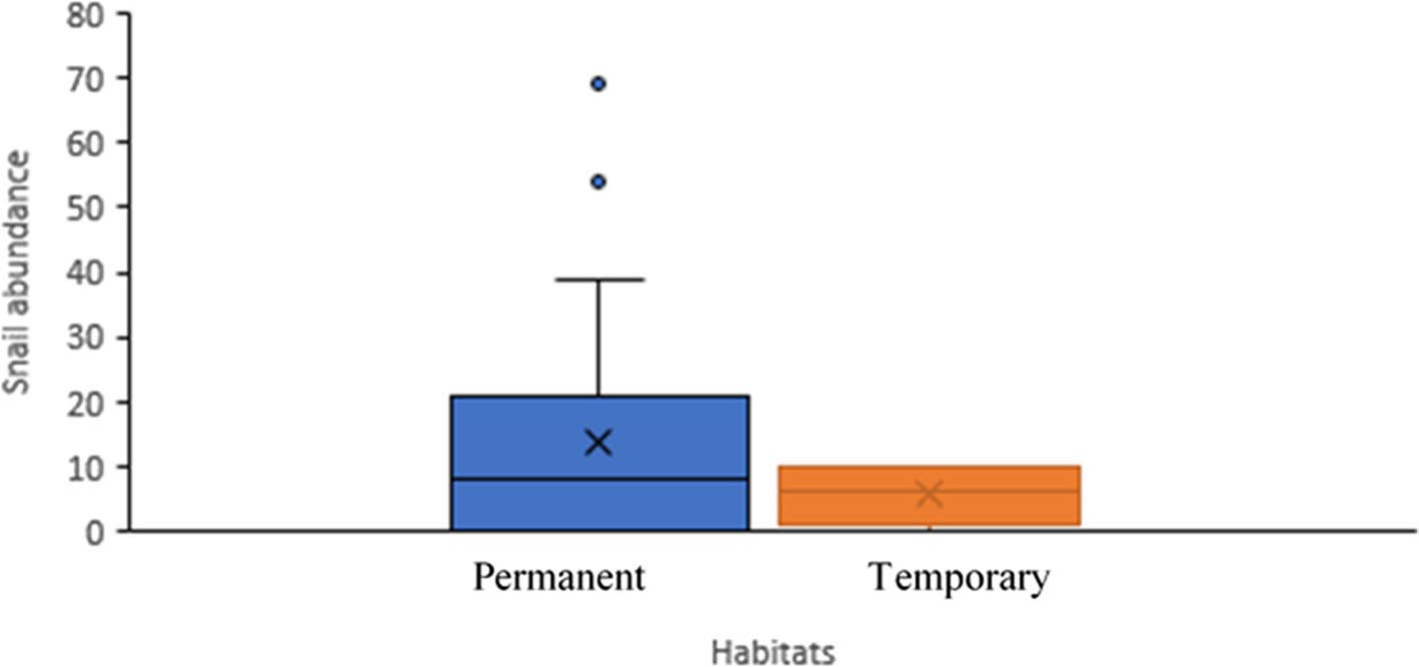
Box and whisker showing the abundance of snails (*P.columella* and *R.natalensis*) in permanent and temporary habitats in uMgungundlovu district

There was a significantly high number of permanent habitats (86.7%; *n* = 39) compared to temporary habitats (13.3%; *n* = 6). The *P. columella* abundance was high (94.2%; *n* = 536) in the permanent habitats and very low (5.8%; *n* = 33) in the temporary habitats (Fig. 3). The box graphically displays the lower quartile (0.25), median (0.5), and upper quartile (0.75); the whiskers depict variability outside the lower and upper quartiles. Wilcoxon test showed no statistical difference between the habitat types (W = 94, *p* = 0.4478).

Table 2 shows the abundance and number of sites of snail species that were encountered during the sampling but were not of interest. *Physella acuta* was found in many sites where *P. columella* was highly abundant while *African mosambuquesis* was found in few sites but interestingly in some of those sites *P. columella* had the most abundance compared to other sites.

**Table 2:** Other snail species encountered during sampling

Species name	Number of sites	Snail abundance
*Physella acuta*	17	286
*Bulinus truncatus*	6	78
*African mosambuquesis*	4	371
*Bulinus globosus*	4	29
*Tarebia granfera*	1	36
*Bulinus Africanus*	3	21
*Radix natalensis*	5	16

Table 3 presents a summary of the water physicochemical parameters from the sampling sites. The water pH values ranged from 6.60 ± 0.38 to 7.46 ± 0.15 (mean ± SD). Dissolved oxygen (DO) varied among the municipalities with uMngeni municipality having the lowest DO value (4.63 ± 1.87 ppm) and uMsunduzi municipality having the highest DO value (7.40 ± 0.57 ppm).

**Table 3:** Physico-chemical values (mean ± SD) measured in seven local municipalities in the uMgungundlovu district between 16 and 26 April 2024

Municipality	pH	Salinity (psu)	Pressure (psi)	Dissolved Oxygen (ppm)	Electrical Conductivity (µs cm^−1^)	Water temperature	
Richmond	7.42 ± 0.49	0.05 ± 0.02	13.56 ± 0.12	6.23 ± 0.64	112.64 ± 38.80		21.78 ± 2.52
Mpofana	7.42 ± 0.62	0.10 ± 0.06	12.47 ± 0.07	4.72 ± 2.04	231 ± 128.89		18.74 ± 3.19
Impendle	6.88 ± 0.433	0.02 ± 0.01	12.34 ± 0.22	6.44 ± 2.53	37.20 ± 14.20		18.75 ± 2.02
uMshwathi	7.46 ± 0.15	0.11 ± 0.08	13.36 ± 0.41	6.50 ± 1.90	248.80 ± 149.31		22.80 ± 1.16
uMngeni	7.18 ± 0.49	0.09 ± 0.09	12.88 ± 0.30	4.63 ± 1.87	160.75 ± 153.41		18.20 ± 2.42
uMsunduzi	6.60 ± 0.38	0.05 ± 8.5^–18^	12.91 ± 0.31	7.40 ± 0.57	105.33 ± 5.03		21.09 ± 1.47
Mkhambathini	7.36 ± 1.02	0.14 ± 0.10	14.05 ± 0.27	6.12 ± 2.64	289.38 ± 208.75		22.51 ± 2.93

### Factors related to the abundance and distribution of *P. columella*

Water temperature, dissolved oxygen, NDWI, pH and water body type had VIF of 5 and below hence were used in the model. Of these that were used in the final model, only, pH showed a statistically significant negative correlation (*p* < 0.05) with *P. columella* abundance (Coeff: −0.478; 95% CI: −0.937— −0.020; *p* = 0.041) (Table 4). The negative binomial generalized linear mixed model used in this study had an AIC of 312.3 compared to poisson model which had AIC of 840.5.

**Table 4:** Summary of properties of environmental exploratory variables for *P. columella* from negative binomial regression GLMM in *“glmmTMB”* package in R studio

Fixed variables	Estimates	Confidence interval		Pr( >|z|)
Water body type	−0.503	−1.436 to 0.430	0.290	
Temperature	−0.071	−0.214 to 0.073	0.334	
pH	−0.478	−0.937 to −0.020	0.041*	
NDWI	−0.014	−0.173 to 0.145	0.864	
Dissolved Oxygen	0.309	−4.310 to 4.928	0.896	

NDWI (Normalized difference wetness index)

^*^Significant correlation at *p* < 0.05

## Discussion

The results of this study show that *P. columella* is widely distributed in uMgungundlovu district with varying abundances within the municipalities. The results also indicate that *P. columella* can thrive in both permanent and temporal habitats making both types of habitats as potential transmission sites [27, 45]. The findings from our study are in consonant with the observations made by Ngcamphalala et al. [33], stated that *P. columella* can thrive in a wide range of freshwater environments, including man-made, natural, temporary, and permanent habitats. The presence of *P. columella* in various habitats indicates that the snail may survive in a variety of environmental conditions within uMgungundlovu, increasing the potential of disease transmission. In addition, the high abundance of *P. columella* across different municipalities may also indicate the invasiveness of the snails that has been observed to be taking over habitats previously known to have native lymnaeid snails [26]. Previously, *R. natalensis* has been found to be highly abundant in many studies [3, 18, 22]. It may thus be reasonable to suggest that *P. columella* outcompetes native snails as it was seen in this study (Table 2), that *R. natalensis* was only present in 5 sites and had very low abundance.

Other snail species may be a sign of the condition of those specific locations because this study indicated that *P. columella* was quite prevalent where other snail species were present. According to Nwoko et al. [34], since biodiversity reflects the range of species and resources that can be found in an ecosystem, it is a crucial indicator of its health. The presents of *P. acuta* and *A. mosambiquensis* could possibly be indicators of the presents of *P. columella*. Invasive *P. acuta* and *P. columella* were found at all sampling sites [40].

The results of our study suggest that pH has a statistically significant effect on the distribution and abundance of *P. columella*. Freshwater snails thrive in environments with pH levels between 6.5 and 8.5 [32]. Suboptimal pH levels enhance the solubility of elements and compounds, hence increasing the mobility of harmful substances. This, in turn, raises the danger of absorption by aquatic organisms, ultimately resulting in mortality. In this study, snails were observed in sites with pH levels between 6.60 and 7.42, which is considered to be the optimal range for surface water [32]. A significant negative association of pH with *P. columella* abundance was recorded in this study. This could be the result of invasive snails (*P. columella*) accelerating nutrient cycling, which alter pH levels by increasing nutrients and total dissolved solids (TDS) in the water column [37]. The results of our study are in agreement with the findings of Nwoko et al. [34], who reported a negative correlation between pH and *Radix natalensis.* Ebenezer and Ekwuribe [18] also reported marginal negative association between pH and *R. natalensis*.

Low dissolved oxygen may be a limiting factor in snail abundance [12]. In the current study the DO ranged from 4.63 to 7.4, which is within the ideal range of freshwater snails (0.4–16.0 ppm) [47]. The DO in our study was positively associated with snail abundance. The positive correlation between dissolved oxygen and snail abundance may be explained by the movement of water, which removes pollutants from the environment making it more conducive for snail survival. The growth of intermediate host snails is also impacted by low oxygen levels in the water and that may explain the reduced snail abundance [39]. Freshwater snails suffocate and perish because of low oxygen content of the water. Boelee and Laamrani [8],Salawu and Odaibo [47] also observed a positive correlation between dissolved oxygen and snail density. In our study, the NDWI was found to be negatively associated with the abundance and distribution of *P. columella*, possibly because habitats were still recovering from rainy season flooding on water bodies where snails reside since sampling was conducted post rain season. Other previous studies observed that NDWI significantly influences the distribution and abundance of intermediate host snails [19, 27]. In the current study, the association between snail abundance and DO was not statistically significant. An earlier observation by Kela et al. [23] and Berrie [7] suggested that some environmental factors may not be statistically significant in influencing that distribution and abundance of snails on their own. However, these authors suggested that such factors may interact with other factors to produce a collective effect, thus leading to the theory of collective effect, as reported by El Deeb et al. [19]. The results observed in the current study partly agree with the observations made by Nwoko et al. [35] who reported marginal negative correlation of NDWI with snail abundance.

The high incidence of Fasciola in South Africa attributed to *P. columella* is a critical concern, characterised by limited knowledge among residents and discrepant prevalence rates in different areas [44]. Emerging evidence suggests that *P. columella* may not only be taking over native *Fasciola* transmission sites but also potentially transmitting both *F. gigantica* and *F. hepatica* in South Africa [25]. This is because the rise in prevalence and incidences of livestock infection with both trematode occurred at the same time *P. columella* was introduced to the country [25]. Therefore, the widespread distribution and significant abundance of *P. columella* across the uMgungundlovu district may be an indication of potential transmission suitability of the area for both *Fasciola* spp. Hadebe et al. [21], stated that, the distribution and abundance of the intermediate host snails are indicators of disease hotspots. A comprehensive understanding of the distribution and significance of *P. columella* in the epidemiology and transmission of Fasciola spp. is essential for accurately forecasting the possible risks and burdens to veterinary and public health.

## Conclusion

Our study demonstrated how *P. columella* is abundant and widely distributed throughout the uMgungundlovu district in diverse environments that coincide with prevalence of fascioliasis. It is therefore important to monitor this snail and understand its population dynamics as it has serious implications on livestock farming in uMgungundlovu. Furthermore, it is important to raise awareness on the spread of *P. columella* among local farmers.

## Data Availability

No datasets were generated or analysed during the current study.
